# Standards for the management of cancer‐related pain across Europe—A position paper from the EFIC Task Force on Cancer Pain

**DOI:** 10.1002/ejp.1346

**Published:** 2019-01-06

**Authors:** Michael I. Bennett, Elon Eisenberg, Sam H. Ahmedzai, Arun Bhaskar, Tony O’Brien, Sebastiano Mercadante, Nevenka Krčevski Škvarč, Kris Vissers, Stefan Wirz, Chris Wells, Bart Morlion

**Affiliations:** ^1^ St Gemma’s Academic Unit of Palliative Care University of Leeds Leeds UK; ^2^ Pain Research Unit, Institute of Pain Medicine Rambam Health Care Campus and Technion, Israel Institute of Technology Haifa Israel; ^3^ University of Sheffield Sheffield UK; ^4^ Imperial College Healthcare NHS Trust London UK; ^5^ Marymount University Hospital & Hospice Curraheen Ireland; ^6^ Cork University Hospital Wilton Ireland; ^7^ College of Medicine & Health University College Cork Cork Ireland; ^8^ Pain Relief and Supportive Care La Maddalena Cancer Center Palermo Italy; ^9^ Faculty of Medicine University of Maribor, Institute for Palliative Medicine and Care Slovenia; ^10^ Department of Anesthesiology, Pain and Palliative Medicine Radboud University Medical Centre Nijmegen Netherlands; ^11^ Centre for Pain Medicine, Department of Anesthesiology, Intensive Medicine, Pain/Palliative Medicine GFO‐Hospitals Bonn and University of Bonn; ^12^ European Pain Federation Brussels Belgium; ^13^ The Leuven Center for Algology and Pain Management University Hospitals Leuven KU Leuven Belgium

## Abstract

**Background and objective:**

Pain is a common symptom in patients who survive cancer and in those who live with progressive advanced disease. Evidence from meta‐analyses suggests that pain remains poorly controlled for a large proportion of patients; barriers to good management include poor assessment of pain, inadequate support for patient self‐management and late or inadequate access to strong opioid analgesia in those with advanced disease.

**Methods:**

The European Pain Federation (EFIC) established a Task Force in 2017 which convened a European group of experts, drawn from a diverse range of relevant clinical disciplines, to prepare a position paper on appropriate standards for the management of cancer‐related pain. The expert panel reviewed the available literature and made recommendations using the GRADE system to combine quality of evidence with strength of recommendation. The panel took into account the desirable and undesirable effects of the management recommendation, including the cost and inconvenience of each when deciding the recommendation.

**Results and conclusions:**

The 10 standards presented are aimed to improve cancer pain management and reduce variation in practice across Europe. The Task Force believes that adoption of these standards by all 37 countries will promote the quality of care of patients with cancer‐related pain and reduce unnecessary suffering.

**Significance:**

Pain affects up to 40% of cancer survivors and affects at least 66% of patients with advanced progressive disease, many of whom experience poor pain control. These 10 standards are aimed to improve cancer pain management, promote the quality of care of patients and reduce variation across Europe.

## 1 INTRODUCTION

Each year, approximately 3.45 million Europeans are diagnosed with cancer of whom 66% will survive for at least 5 years and 40% will be alive more than 10 years after their diagnoses (Ferlay et al, 2013; Glare et al, 2014).

Pain is the commonest symptom of cancer at diagnosis (Breivik et al, 2009) and rises in prevalence throughout and beyond cancer treatment (van den Beuken‐van Everdingen et al, 2016). Between 33% and 40% of cancer survivors (persons with cancer whose curative treatment was completed) suffer from chronic pain (Boland and Ahmedzai, 2017; Paice, 2011; Seretny et al, 2014). In contrast, 1.7 million European cancer patients die from their disease each year of whom at least 66% will experience pain before death and 55% will experience moderate‐to‐severe intensity pain (Ferlay et al, 2013; van den Beuken‐van Everdingen et al, 2016).

It is more than 30 years since the publication of the WHO method or guidelines for cancer pain relief (WHO, 1996). Despite this and the publication of European guidelines (Caraceni et al, 2012; Raphael, Ahmedzai, et al, 2010; Ripamonti et al, 2012; Swarm et al, 2013), there remain barriers to good control of cancer‐related pain (Oldenmenger et al, 2009). At least one‐third of patients are undertreated which is often due to inadequate attention to pain during regular oncological treatment, and inequitable or delayed access to opioids (Gagnon et al, 2015; Greco et al, 2014; Te Boveldt et al, 2015; Ziegler et al, 2016). Uncontrolled or undertreated pain may become physically and emotionally disabling, leading to increased suffering and reduced quality of life (Te Boveldt et al, 2013).

A Task Force was sourced by the European Pain Federation (EFIC) in 2017. In line with other standards development groups, a panel of international experts was instituted, chaired by EE and MB. Experts from European countries were invited by email to participate, explaining the rationale for the process, and aiming for broad geographical representation within Europe.

EFIC recognizes that improving the experience of patients with cancer‐related pain requires actions that focus on patients, clinicians and healthcare providers or systems. The EFIC Task Force on cancer‐related pain reviewed existing evidence‐based guidelines and reached a consensus on the standards for the management of cancer‐related pain across all 37 European countries that are members of the European Pain Federation (EFIC). This paper describes 10 standards that EFIC member countries should institute or be working towards. The standards do not specify which healthcare professional(s) should conduct assessments, agree management plans, provide tailored treatment and support, or refer for more specialized advice and treatment. The Task Force considered that all services involved in the care of patients with cancer should consider how best to meet these standards considering local expertise and resources. These standards may also prompt greater collaboration between clinicians in pain medicine, palliative care and oncology to meet the needs of patients with cancer‐related pain.

The GRADE (Grading of Recommendations Assessment, Development and Evaluation) system was used to determine recommendations for each standard based on quality of evidence and strength of recommendation (Guyatt et al, 2008). Recommendations were judged by the Task Force based on weighing up the desirable and undesirable effects of the management recommendation, including the cost and inconvenience of each. Each Task Force member was asked to assign a recommendation for each standard, and the final recommendation was based on majority consensus where agreement was not unanimous (Box 1).

BOX 1 **GRADE recommendations (Guyatt et al., 2008)**

**Grade Recommendations**

1A. Strong recommendation, high‐quality evidence

1B. Strong recommendation, moderate‐quality evidence

1C. Strong recommendation, low‐quality evidence

2A. Weak recommendation, high‐quality evidence

2B. Weak recommendation, moderate‐quality evidence

2C. Weak recommendation, low‐quality evidence

## 2 STANDARDS

### 2.1 Standard 1. Patients with a history of cancer should be routinely screened for pain at every engagement with a healthcare professional. [GRADE 1B]

Supporting patients with cancer‐related pain requires systematic screening for pain symptoms for both patients in remission and those with ongoing disease (Swarm et al, 2013; Trowbridge et al, 1997; Williams et al, 2015). Patients are often reluctant to disclose pain as they do not perceive healthcare professionals are interested or have the time, and these attitudes are often consolidated because healthcare professionals rarely ask about pain routinely (Gibbins et al, 2014). Screening for pain can be achieved by using routine questions in consultations; for example “Have you experienced pain that interferes with daily activities?,” or by use of paper or electronic questionnaires in clinics or on hospital wards, such as Numerical Rating Scales (NRS) or Visual Analogue Scales (VAS).

### 2.2 Standard 2. Patients identified with cancer‐related pain should receive a pain assessment when seen by a healthcare professional, which at a minimum classifies the cause of pain based on proposed ICD‐11 taxonomy and establishes the intensity and impact on quality of life of any pain that they report. [GRADE 1B]

Cancer‐related pain has multiple aetiologies including the cancer itself (cancer pain) and cancer treatments, particularly surgery, chemotherapy (including hormonal, biological and immune therapies) and radiotherapy (Bennett, 2017). It can originate from visceral, bone or nerve tissues and can have nociceptive, neuropathic or inflammatory mechanisms (Falk and Dickenson, 2014; Knudsen et al, 2009). It also varies in its temporal characteristics: it can be acute or chronic and may have continuous or episodic features (Caraceni and Portenoy, 1999). Persistent cancer pain can lead in some individuals to the development of chronic widespread pain induced by plastic changes in the somatosensory nervous system) Kosek et al, 2016).

Pain may also be caused by comorbid conditions unrelated to cancer, and this aetiology accounts for around 10%–20% of pains in cancer patients (Bennett et al., 2012; Grond et al., 1996). Therefore, pain in a cancer patient is not synonymous with cancer‐related pain; a clinical assessment must distinguish between cancer pain, cancer treatment pain and pain from comorbid conditions.

Often, patients experience mixed types of pain simultaneously, or pain that changes over time. A detailed diagnostic assessment that must be repeated at appropriate intervals is therefore often required to guide treatment strategies.

A bedside assessment can determine the intensity, aetiology, character and underlying mechanisms of pain leading to improvements in pain outcomes (Trowbridge et al, 1997). The new proposed ICD‐11 classification for cancer‐related pain (Table 1) will enable a standardized taxonomy for clinical practice and research and should be used widely (Bennett et al., 2019; Treede et al, 2015). The Brief Pain Inventory provides a summary of the severity and impact of the pain on the patient’s daily activities (Cleeland and Ryan, 1994). Additionally, conditions which can amplify pain expression such as distress, anxiety and depression (Laird et al, 2016), delirium or effects of alcohol and mis‐used drugs, should be incorporated in a structured pain assessment (Table 2).

**Table 1:** Proposed ICD‐11 classification of cancer‐related pain

Level 1	Level 2	Level 3	Level 4
Cancer‐related pain	Cancer pain	Visceral cancer pain	
		Bone cancer pain	
		Neuropathic cancer pain	
	Postcancer treatment pain	Postcancer medicine pain	Painful chemotherapy‐induced polyneuropathy
		Postradiotherapy pain	Painful radiation‐induced neuropathy
		Postcancer surgery pain	

**Table 2:** Structured pain assessment

History	Pain location, onset, duration, severity, quality, alleviating and aggravating factors. Special emphasis on episodic pain
	Impact on mood, usual activities, function, quality of life and sleep
	Previous pain and treatment history
	Ongoing response to treatment, adverse effects
	Comorbidities impacting pain (e.g., chronic disease, surgery, trauma, mood, cognitions, substance use disorder, medications)
	Personal characteristics (e.g., age, sex, race, religion, culture, language)
	Expectations of pain management and current understanding of the condition, including what significance the patient attaches to the pain
Physical examination (focused according to the presenting condition)	Relevant, physical, neurological and musculoskeletal assessment
Review of clinical records	Comorbid diseases, previous chronic pain history, age‐related frailty
Investigations	Laboratory tests
	Imaging studies Neurophysiological evaluations

### 2.3 Standard 3. A multimodal pain management plan should be agreed with the patient who explains the causes of their pain and its likely prognosis, the need for further investigations, the multimodal treatment options, and includes the patient’s preferences and goals for treatment. [GRADE 1C]

Cancer patients with pain are keen to understand the cause of their pain, what to expect, options for pain control, and how to cope with cancer pain including talking with others and finding help (Bender et al, 2008). Longitudinal interview studies reveal that pain is very dynamic and complex for patients and that pain control is often a trial and error process that requires continuous work (Hackett et al, 2016). Cognitive and other side effects of analgesia for cancer‐related pain impact on quality of life (Sloot et al, 2015). Patients often try to manage by reducing interference from both pain and these side effects to maintain as much function as possible. This “trading‐off” between pain and side effects can impact on medication adherence and should be acknowledged within the pain management plan (Flemming, 2010; Manzano et al, 2014).

The goals of cancer‐related pain management should therefore be to reduce the pain and its impact on daily living though tailored treatment, and to increase each patient’s ability for self‐management (Bennett, 2017; Gibbins et al, 2014). Helping patients to achieve this balance and identifying realistic expectations of treatments (for example that pain may be controlled to an acceptable degree rather than relieved completely) are important for improved patient outcomes.

The care of many patients with cancer may be provided by different healthcare professionals simultaneously, for example a surgeon, oncologist or primary care physician. It is therefore essential that all healthcare professionals who are involved in the care of patients with cancer‐related pain be appropriately trained to complete this assessment, to initiate evidence‐based treatment and to refer to a competent specialist when needed (see standard 7). However, most patients can be adequately treated at a nonspecialized care level and on an outpatient basis. Proper coordination is required between the different healthcare professionals to ensure consistent and quality care.

### 2.4 Standard 4. Patients should receive tailored multimodal treatment which reduces the pain and its impact on daily living and that may include a combination of medicines, nonpharmacological treatments, oncological interventions, physical rehabilitation and psychosocial or spiritual support. [GRADE 1A]

Patients must have access to drugs and other interventions which are tailored to them. This means that the treatments are believed to be effective in their type of cancer‐related pain, and that they are consistent with the patient’s preferences and goals for treatment.

Analgesic medication that is appropriate for the patient and their pain should be available within 24 hr of a pain assessment where indicated; this must include access to a prescriber as well as access to a dispensed prescription (Table 3).

**Table 3:** Essential analgesic drugs

Drug class	Examples
Simple analgesia	Paracetamol Nonsteroidal anti‐inflammatory drugs (NSAIDs) including coxibs
Opioids	*Opioids for moderate intensity pain*: Codeine, dihydrocodeine, tramadol *Opioids for moderate to severe intensity pain*: Immediate release opioids as oral and injectable formulations (morphine, oxycodone, hydromorphone); Sustained release opioids as oral (morphine, oxycodone, tapentadol, hydromorphone;) and transdermal (fentanyl, buprenorphine) formulations, *Opioids for specialist use only* Rapid onset transmucosal fentanyl based formulations Methadone
Antidepressants	Amitriptyline, imipramine, duloxetine, venlafaxine
Antiepileptics	Gabapentin, pregabalin, carbamazepine
Corticosteroids	Prednisolone, dexamethasone
Bisphosphonates	Pamidronate, Zoledronate
Monoclonal antibodies	Denosumab, Tanezumab
Other	Topical lidocaine; ketamine *(specialist use only)*

Prescribers should adhere to regional or national protocols or evidence‐based guidelines for cancer‐related pain (Caraceni et al, 2012; Raphael, Hester, et al, 2010; Ripamonti et al, 2012; Swarm et al, 2013) as this improves outcomes for patients (Du Pen et al, 1999, Cleeland et al, 2005).

Although analgesic medications are a cornerstone in the management of cancer‐related pain, the use of other modalities and procedures can often improve outcomes.

Access to opioid analgesia is critical for pain related to advanced and progressing cancer.

For patients with cancer‐related pain who are in the last weeks or days of life, a tailored treatment plan is especially important and should anticipate analgesic needs (NICE, 2015). Medications aimed to treat opioid‐related side effects such as laxatives, peripheral opioid antagonists and antiemetics should be readily available.

Opioid analgesia may not always be suitable for long‐term management of pain related to cancer treatments (chemotherapy or surgery) in the context of disease that has been cured or is in remission (Boland and Ahmedzai, 2017; O’Brien et al., 2017).

Access to oncological and surgical management options to control pain should be available regionally within each country. For example, radiotherapy for bone metastases, spinal stabilization, surgical fixation of pathological fractures, vertebroplasty or palliative debulking of tumour.

### 2.5 Standard 5. Support and advice for self‐management should be provided [GRADE 1A]

Self‐management is the capability of a patient to manage their pain, their analgesic treatments and the physical and psychological consequences of living with cancer‐related pain (Johnston et al, 2014). This involves a number of skills and activities including getting the right information, managing practical tasks and emotions, solving problems and knowing what to do when symptoms get worse or more help is needed (Schumacher et al, 2014). Most patients want help to manage themselves, but their ability to do this is influenced by their emotional state and their physical health.

Addressing concerns about pain and strong opioids, and providing strategies that help patients though provision of educational materials, improve patient outcomes and should be routine practice (Bennett et al, 2009; Oldenmenger et al, 2018; Sheinfeld Gorin et al., 2012). This information must cover how to take analgesia, the likely effectiveness of this, how to monitor side effects, plans for further follow‐up, and how to get help especially out of hours. All members of the healthcare team have a role in supporting self‐management including doctors, nurses pharmacists and others.

### 2.6 Standard 6. The pain management plan should be reviewed regularly to assess outcomes and plan longer‐term care [GRADE 1B]

Although patients understandably express that they want to be pain free, in general, they do not actually expect their pain to go completely (Gibbins et al, 2014). Most patients seem to determine whether their pain is controlled by whether or not they can perform activities or tasks and maintain relationships with family or friends. The outcome in terms of the balance of residual pain and functioning is individually determined.

Patients should know who is responsible for reviewing their pain management plan, when this review is expected to take place and whether this can be done face‐to‐face, by telephone or digital technology (Adam et al., 2017). Contact information for out of hours support should be easily accessible. Telephone support from a nurse or pharmacist integrated with electronic symptom monitoring is an effective method of follow‐up for pain and analgesia (Kroenke et al, 2010; Oldenmenger et al, 2011).

### 2.7 Standard 7. Patients should be referred for more specialist advice and treatment if pain is not improving within a short time or if they are experiencing intolerable side effects of analgesia [GRADE 1C]

Patients should be referred for specialist support if pain is not well controlled despite initial management or the pain has been identified as complex. Specialist support must be available regionally within each country in the form of specialist multidisciplinary pain services, oncology services including radiotherapy and palliative care services. Access to specialist services for pain that is insufficiently responding to standard care should be readily available regionally within one week (IASP, 2018). This includes access to advanced pain management techniques such as intrathecal pumps and neuro‐ablative techniques (Bruel and Burton, 2016; Smith et al, 2002; Vayne‐Bossert et al, 2016). Early referral to specialist services is needed when pain is complex or not responding to initial treatment, rather than waiting until all conventional approaches have been exhausted and the patient is too unwell for advanced pain management intervention.

Patients who present with severe uncontrolled pain, significant drug related adverse events or extreme psychological distress should also be referred urgently to a specialized care facility (IASP, 2018). Healthcare professionals should be aware of appropriate specialized care facilities with specific and targeted multidimensional expertise in the management of cancer pain nationally. Established referral pathways should exist between oncology, pain and palliative care services to coordinate care while taking into account patient and family expectations.

With improved early cancer diagnosis and enhanced treatments, many patients are now living with cancer as a chronic disease. Increasing numbers of patients experience cure or prolonged remission and are regarded as cancer survivors. Some of these patients will continue to experience pain which negatively affects their quality of life (Glare et al, 2014). Some patients may continue to use high doses of opioid, previously required for adequate pain control, but which are no longer needed while causing side effects. These patients require more specialist help which is likely to include long‐term self‐management plans, medication weaning plans and pain rehabilitation and occupational therapy interventions (Paice et al, 2016). Such plans are typically offered by chronic pain management programs and pain clinicians with a special interest in opioid management. Each country should have national guidance on managing and referring patients with long‐term pain from cancer treatments.

### 2.8 Standard 8. Healthcare professionals who treat patients with cancer should receive ongoing education and training in order to undertake basic pain assessment, initiate basic management, and learn about correctly referring for more specialist support [GRADE 1C]

Significant gaps in knowledge on cancer pain diagnosis and management by healthcare professionals are well documented in the medical literature, leading to inadequate relief in a substantial number of patients with cancer pain (Oldenmenger et al, 2009). Proper education and training is therefore essential. EFIC recommends its chapters to develop educational programs on cancer pain at undergraduate level (medical and nursing schools), postgraduate level for all healthcare professions who are involved in the care of patients with cancer, and finally within specialized programs within training in pain and palliative medicine. An example of the contents of such educational programs can be found in EFIC’s Core Curriculum for Pain Management (European Pain Federation, 2017).

### 2.9 Standard 9. Regular review of service outcomes for all patients with cancer pain should be in place [GRADE 1C]

Regular review of outcomes for patients enables services to identify areas for improvement, focusing on safety and efficacy data of various methods of pain control. Novel methods for supporting patients to complete outcome measures are needed, for example using electronic or digital technology (Adam et al., 2017).

### 2.10 Standard 10. Each EFIC chapter should have national evidence or consensus‐based guidelines in place for cancer‐related pain [GRADE 1C]

Many international and national guidelines on cancer pain assessment and treatment are available but they may not be suitable for all countries (Piano et al, 2014). EFIC recommends that its chapters should produce an appropriate treatment guideline for cancer‐related pain that is valid for the circumstances according to each country's needs acknowledging variations in access to drugs and other treatments, and respecting social and cultural identity. Such guidelines can be adapted from existing international or national guidelines in other countries. Production of lay versions which can be understood by nonhealthcare professionals should be considered.

We recommend that each country will develop a mechanism, by either professional organizations or through regulatory authorities, to monitor and assess the implementation and usage of national guidelines and European standards.

## 3 SUMMARY

The 10 standards presented are aimed to improve cancer pain management and reduce variation across Europe. We believe that adoption of these standards by all 37 countries will promote the quality of care of patients with cancer‐related pain and reduce unnecessary suffering. EFIC will continuously support enhancing the quality of cancer pain management through its pain schools, special sessions in conferences and reviewing and when necessary revising this document in the future.

## AUTHOR CONTRIBUTIONS

Each of the authors made a substantial contribution to the development of the paper by informing its content, collaborating in the drafting of the various sections and reviewing the final draft. MB and EE served as chairs of the group, wrote the first draft and coordinated the editing of the final draft.
